# Editorial of Special Issue “Regulatory Roles of Inflammasomes in Human Diseases”

**DOI:** 10.3390/ijms22063008

**Published:** 2021-03-16

**Authors:** Young-Su Yi, Miyong Yun

**Affiliations:** 1 Department of Life Sciences, Kyonggi University, Suwon 16227, Korea; 2 Department of Bioindustry and Bioresource Engineering, Sejong University, Seoul 05006, Korea; myyun91@sejong.ac.kr

**Keywords:** inflammasome, inflammation, human disease, inflammasome-targeted therapy

## Abstract

Inflammation is an innate immunity protecting the body from pathogens and cellular damages and comprises two steps; 1) priming (preparatory step) and triggering (activation step). The key feature of the triggering step is the activation of inflammasomes that are intracellular protein complexes consisting of pattern recognition receptors and inflammatory molecules. Inflammasomes are activated in response to various ligands, leading to the caspase-1-mediated maturation and secretion of pro-inflammatory cytokines, IL-1β and IL-18 and the gasdermin D-mediated pyroptosis, an inflammatory form of cell death. Previous studies have demonstrated that inflammasome activation is a key determinant of inflammatory responses and many human diseases; therefore, inflammasomes have been attracted much attention as critical drug targets to prevent and treat various human diseases.

In this Special Issue, we invited the original and review articles investigating the mechanisms of inflammasome regulation, roles of inflammasomes in human diseases, identification/validation of novel molecular targets modulating inflammasome functions and development of inflammasome-targeting strategies and therapeutics and three research and three review articles that investigated the roles of inflammasomes in human diseases were published.

The first research article by Kwak et al., investigated the reno-protective effect of erythropoietin (EPO), a stimulator of erythroid progenitor cells, in an ischemia/reperfusion-induced acute kidney injury (IR-AKI) by modulating inflammasome function. This study demonstrated the ameliorative effect of EPO in an IR-AKI mouse model via suppressing NLRP1 and NLRP3 inflammasome activation [1], which suggests that inflammasome-induced inflammation could be a potential target of EPO to treat IR-AKI.

The next research article by Cyr et al., reported ASC, IL-18 and galectin-3 as biomarkers of non-alcoholic steatohepatitis (NASH), a severe form of non-alcoholic fatty liver disease. This study is a proof of concept study of biomarker analyses on NASH patient sera and demonstrated that the serum levels of ASC and IL-18 that are inflammasome-associted molecules and galectin were significantly elevated in the NASH patients [2], which suggests that inflammasome components are reliable molecular biomarkers of NASH that could increase the diagnostic potential for NASH.

The final research article by Mezzasoma et al., investigated the molecular mechanism by which natriuretic peptides (NPs) play an anti-inflammatory effect via modulating inflammasome function in the monocytes, THP-1 cells during the inflammatory responses. This study demonstrated that both atrial natriuretic peptides (ANP) and B-type natriuretic peptides (BNP) suppressed the inflammatory responses by inhibiting NLRP1, NLRP3 and AIM2 inflammasome activation and interfering with gasdermin D and IL-1 activation in THP-1 cells [3]. These results show that ANP and BNP play anti-inflammatory and immunomodulatory roles via suppressing inflammasome activation in monocytes and provide the potential of NPs as anti-inflammatory agents to treat various human diseases caused by dysregulation of inflammasome activation.

The first review article by Yi summarized and discussed the current studies exploring the activation mechanisms and the regulatory roles of non-canonical inflammasomes, such as mouse caspase-11 and human caspase-4 and caspase-5 non-canonical inflammasomes in the inflammatory response and human diseases [4]. This review provided insight into new strategies for the development of anti-inflammatory therapeutics against infectious and inflammatory diseases via targeting non-canonical inflammasomes.

Interestingly, the second review article by Wagatsuma and Nakase comprehensively summarized the regulatory mechanisms underlying the contradictory effects of NLRP3 inflammasome in colitis. This review discussed the literature on the functional crosstalk between NLRP3 inflammasome and inflammatory cytokines, autophagy and microbiota in colitis animal models and inflammatory bowel disease patients and provided new insight into the different and contradictory roles of NLRP3 inflammasome and the underlying regulatory mechanisms in colitis [5].

The final review article by Yi discussed the anti-inflammatory and ameliorative role of flavonoids in rheumatic diseases by inhibiting inflammasome activation. Flavonoids are natural compounds found throughout the plant kingdom that have various biological and pharmacological activities, including anti-inflammatory activity [6]. Despite the anti-inflammatory activity of flavonoids, the underlying molecular mechanisms have not been fully understood and much effort has been made on deciphering the molecular mechanisms of the flavonoid-mediated anti-inflammatory effect by modulating the inflammasome functions. This review summarized and discussed the studies investigating the anti-inflammatory and ameliorative effect of various flavonoids in rheumatic diseases via inhibiting inflammasome activation. This review further provided the potential of flavonoids as nutraceuticals to prevent and treat various inflammatory and autoimmune diseases with new mechanisms that target inflammasome activation.

We hope that the readers are interested in understanding the regulatory roles of inflammasomes in not only rheumatic diseases but also many human inflammatory and autoimmune diseases. Additionally, we also hope this special issue attracts the attention and interest of the scientific community, thereby further contributing to future researches discovering new roles of inflammasomes in human diseases and developing new therapeutics for the prevention and treatment of human diseases.
